# Does progesterone have protective effects on ovarian ischemia-reperfusion injury?

**DOI:** 10.4274/jtgga.2017.0047

**Published:** 2018-06-04

**Authors:** Banu Güleç Başer, Mine İslimye Taşkın, Ertan Adalı, Emine Öztürk, Adnan Adil Hısmıoğulları, Arzu Yay

**Affiliations:** 1 Department of Obstetrics and Gynecology, Balıkesir University School of Medicine, Balıkesir, Turkey; 2 Department of Biochemistry, Balıkesir University School of Medicine, Balıkesir, Turkey; 3 Department of Histology and Embryology, Erciyes University School of Medicine, Kayseri, Turkey

**Keywords:** Ischemia-reperfusion, NGAL, progesterone

## Abstract

**Objective:**

The aim of the present study was to evaluate the effects of progesterone (PG) against ovarian ischemia-reperfusion (I/R) injury through the evaluation of biochemical and histopathologic parameters.

**Material and Methods:**

Twenty-one female Wistar albino rats were divided into three groups. Group 1: Sham; group 2: I/R; group 3: I/R+PG (8 mg/kg). PG was administered intraperitoneally to the rats in group 3, 30 minutes before a detorsion operation. Ovarian I/R injury was evaluated in serum and tissue by using biochemical parameters including malondialdehyde (MDA), total antioxidant status (TAS), total oxidant status (TOS), oxidative stress index, neutrophil gelatinase-associated lipocalin (NGAL) and immunofluorescence staining by using a terminal deoxynucleotidyl transferase-mediated dUTP nick end labeling (TUNEL) assay.

**Results:**

Serum and tissue TOS levels were significantly lower in group 3 than in group 2. Tissue TAS levels were higher in group 3 than in group 2 (p<0.001). NGAL and MDA levels were similar between the groups. Histologic score, including vascular congestion, hemorrhage, polymorphonuclear neutrophils, and interstitial edema, was higher in group 2. Pre-treatment with PG decreased the score, but this difference was not statistically significant. The number of apoptotic cells was higher in group 2 than in groups 1 and 3. The TUNEL-positive cell number decreased with PG in group 3.

**Conclusion:**

Preoperative PG treatment might exert protective effects on ovarian I/R injury through its anti-apoptotic and antioxidative properties.

## Introduction

Adnexal torsion is a serious cause of gynecologic emergency surgery, with a prevalence of 2.7%. Diagnosis and intervention are delayed because clinical findings in adnexal torsion are nonspecific. This can lead to a decrease in the patient's follicle reserve and infertility. Excision of the adnexa, detorsion of the pedicle, and evaluation of tissue perfusion are considered and conducted as the conservative approach today (1). After the detorsion procedure, there are increases in neutrophil infiltration and activation, production of nitric oxide and cytokines such as tumor necrosis factor, and superoxide radicals (SOR) in ovarian tissue. This condition, called ischemia-reperfusion (I/R) injury, causes more tissue damage than ischemia alone. Therefore, it has been suggested that the use of antioxidant pharmacologic agents during or prior to reperfusion may be beneficial in protecting against I/R injury (2).

Protective effects of progesterone on traumatized neurons, heart, and vascular tissues have been demonstrated. The focus of these studies is the suppressive effect of progesterone on the inflammatory process (3,4,5,6). It has been experimentally determined that progesterone reduces SOR formation and prevents cellular damage through examining levels of malondialdehyde (MDA) and glutathione (GSH), which are lipid peroxidation products in the liver and kidney, after benzene-induced toxicity (7). Experimental studies have shown that PG suppresses SORs and their oxidative damage. It also has a protective effect against lipid peroxidation and cell membrane damage due to free radicals (8). It has been also shown in another study that progesterone reduces MDA and myeloperoxidase levels, significantly increases GSH and superoxide dismutase levels, and significantly suppresses apoptosis (9). All these studies suggest that progesterone may also have a protective effect on ovarian tissue.

This is the first experimental study to examine the effect of progesterone on ovarian I/R injury. For this purpose, we investigated whether progesterone caused changes in biochemical, histologic, and immunohistochemical markers in rat ovaries exposed to I/R. 

## Material and Methods

This study was conducted with the consent of the Animal Experiments Ethics Committee and “Principles of laboratory animal care” (NIH publication no: 86-23, revised 1985) were followed, as well as specific national laws where applicable. A total of 21 young adult female Wistar albino rats weighting 200-250 g were used in the study. The experimental animals were housed on a 12-hour light/dark schedule under a temperature of 21-22 °C with ad libitum feeding. Group 1: sham group (n=7); group 2: I/R, applied for 3 hours with torsion, and for 3 hours after detorsion, and saline intraperitoneally 30 minutes prior to detorsion (n=7); group 3: I/R+PG, administered for 3 hours with torsion, and for 3 hours after detorsion, and 8 mg/kg progesterone intraperitoneally 30 minutes prior to detorsion (n=7) (10). The rats were anesthetized using ketamine 75 mg/kg and xylazine 10 mg/kg intraperitoneally (11). They underwent laparotomy with a 2-cm midline incision and the right and left adnexa of all rats were torsioned for 3 hours, except for the control group. The torsion procedure was performed by rotating both adnexa 360 degrees clockwise. The torsioned adnexa was fixed to the abdominal wall muscles with 4/0 Vicryl, and the skin was also sutured with 3/0 Vicryl. Sham operations (placebo surgery) were performed on group 1, undergoing laparotomy only. Thirty minutes prior to detorsion, the rats in group 2 were injected with saline and the rats in group 3 were injected with progesterone intraperitoneally. At the end of the 3-hour torsion period, the rats were anesthetized again and underwent laparotomy via the same incision line. The detorsion procedure was then performed counterclockwise. At the end of the detorsion period, intracardiac blood samples were taken from all rats before they were sacrificed. After the rats were sacrificed, the right ovary was taken for biochemical analysis and the left ovary was taken for histopathologic analysis.

### Biochemical analysis

Serum was separated by centrifuging whole blood at 825×g for 10 min and subsequently used for analyses of total protein, total antioxidant status (TAS), and total oxidant status (TOS) using commercially available kits in an autoanalyzer (Cobas Integra 800; Roche Diagnostics GmbH; Mannheim, Germany). Serum and ovarian TAS and TOS levels were measured spectrophometrically (PerkinElmer’s Lambda 35 UV/Vis, USA) using a commercially available kit (Rel Assay Diagnostic, Turkey). The novel automated TAS method is based on antioxidants in the sample reducing dark blue-green colored 2, 2'-Azino-bis (3-ethylbenzothiazoline-6-sulfonic acid) (ABTS) radical to the colorless reduced ABTS form. The TOS assay is based on oxidants present in the sample that oxidize the ferrous ion chelator complex to ferric ion (12). The assay was calibrated with hydrogen peroxide, and the results are expressed in terms of micromolar hydrogen peroxide equivalent per liter (*μ*mol H_2_O_2_ Equiv./L) for serum and hydrogen peroxide equivalent per gram protein (*μ*mol H_2_O_2_ Equiv./g protein) for ovarian extracts.

The ratio of TOS to TAS was accepted as the OSI. In order to calculate this, the resulting unit of TAS was converted to µmol/L, and the OSI value was calculated using the following formula as previously published elsewhere (12):

OSI (arbitrary unit)=TOS (*μ*mol H_2_O_2_ Equiv./L)/TAS (mmol Trolox equivalent/L)

Serum MDA levels were determined based on the spectrophotometric measurement of the product generated upon the reaction of MDA with thiobarbituric acid. The results are expressed as *μ*mol/mL in serum and *μ*mol/g protein in tissue.

Serum neutrophil gelatinase-associated lipocalin (NGAL) measurement was conducted using SUNREDBIO-Human NGAL enzyme-linked immunosorbent assay kit 96 test according to guidelines in the kit guide.

### Immunohistochemical analysis

After the rats were sacrificed, excised ovarian tissues were stored in 10% formaldehyde for fixation. The ovarian tissues were kept in fixation solution for 72 hours and then washed with running tap water. They were passed through graded alcohol series and cleared using xylol. Then they were embedded into paraffin and blocked. Sections of 5 *μ*m were taken from the ovarian tissues and placed onto polylysine-coated laminas. Using a standard histologic follow-up method, the paraffin of the laminas were removed with xylol, and the laminas were passed through graded alcohol series (100%, 96%, 80%, 70%, 50%) and washed with water. In order to determine the general histologic structure, the sections were stained using hematoxylin-eosin and Masson’s trichrome, passed first through increasing alcohol series and then through xylol, and covered with a lamella using Entellan.

Follicular cell injury, hemorrhage, vascular congestion, and polymorphonuclear cell infiltration were evaluated in a histopathologic evaluation of ovarian injury. Semi-quantitative scoring was conducted using a value between 0-3 according to injury scoring of 0: no damage, 1: slightly damaged, 2: moderately damaged, and 3: severe (13).

A terminal deoxynucleotidyl transferase (TdT)-mediated dUTP nick end labeling (TUNEL) assay was used in order to determine apoptotic cells in all sections of tissues obtained at the end of the experiment. An ApopTag^®^ Fluorescein *in situ* Apoptosis Detection Kit (EMD Millipore, Darmstadt, Germany) was used for staining. Ovarian tissues of 5-6 *μ*m thickness were deparaffinized and rehydrated (absolute alcohol, 96%, 80%, 70%, 60%, and 50%), and then washed 3 times with PBS. Slides were incubated with proteinase K for 15 min, then washed with distilled water. The samples were treated with 3% hydrogen peroxide for 10 min to minimize endogenous peroxidase activity. The tissues were washed 3 times with PBS for 5 min each and then incubated with a TUNEL reaction mixture from the kit for 1 hour in humid and dark environment at 37 °C. The tissues were stained with contrast dye using 4',6-diamidino-2-phenylindole to observe the nuclei. All operations were performed in a humid chamber. The same procedures were conducted on the tissue used as a negative control but without adding TdT. The prepared samples were evaluated using a fluorescence microscope (Olympus BX51, Tokyo, Japan). To assess the number of TUNEL-positive apoptotic cells, at least five different areas were photographed on each tissue using a 40x lens. After the immunofluorescence staining procedure, positively-stained apoptotic cells were carefully counted using the Image J software program.

### Statistical analysis

Statistical analyses were performed using SPSS v. 11.5. Data are shown as mean ± standard deviation, median (IQR) or median (minimum-maximum), as appropriate. For normally distributed data, the mean differences among groups were analyzed using one-way ANOVA and for the remaining data, the Kruskal-Wallis test was used for comparisons of the medians. A p-value less than 0.05 was considered statistically significant. When the p value from one-way ANOVA or Kruskal-Wallis test statistics were statistically significant, post hoc Tukey’s honestly significant difference or Bonferroni-adjusted Mann-Whitney U test was used to specify which group significantly differed from the others.

## Results

The serum TOS level was significantly higher in the I/R group compared with the sham group (p<0.001). The serum TOS level was found to be significantly lower in the I/R+PG group compared with the I/R group (p<0.001). The serum OSI level increased in the I/R group compared with the sham group, but decreased significantly in the group treated with progesterone (p<0.001) Serum MDA and NGAL levels decreased in the group treated with progesterone, although the differences were not statistically significant (Table 1).

There was a statistically significant difference among groups in terms of tissue TOS levels; the tissue TOS level of I/R group was higher than in the sham group (p<0.001). Progesterone treatment was observed to result in a decrease in tissue TOS levels compared with the I/R group (p<0.001). The tissue TAS level was increased in the group treated with progesterone after I/R (p<0.05). The mean tissue OSI level was significantly higher in the I/R group compared with the sham and I/R+PG groups (p<0.001). The OSI level was decreased in the I/R+PG group compared with the I/R group (p<0.001) (Table 2). 

**Figure 1:**
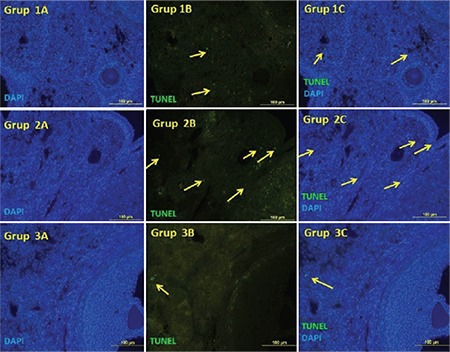
Apoptotic cells are stained with terminal deoxynucleotidyl TUNEL in groups. TUNEL-positive cells reflective green immunofluorescence. Positive apoptotic cells were counterstained with DAPI nuclear staining. There were many apoptotic cells in group 2. There were only a few TUNEL-positive cells in the ovaries of group 3 (Original magnification, 400x) *DAPI: 4',6-diamidino-2-phenylindole, TUNEL: Transferase-mediated dUTP nick end labeling*

There was a statistically significant difference between the groups in terms of histopathologic score. The median histopathology score of the I/R group was higher than that of the sham group (p<0.001). The histologic score was found to be lower in the I/R+PG group compared with the I/R group, although it was not statistically significant (Graphic 1). Considering apoptotic cell numbers, the number of ovarian TUNEL-positive cells in the I/R group was higher than in the sham group (p<0.001). In the group treated with progesterone, the number of TUNEL-positive cells decreased in the I/R group (Figure 1).

**Figure 2:**
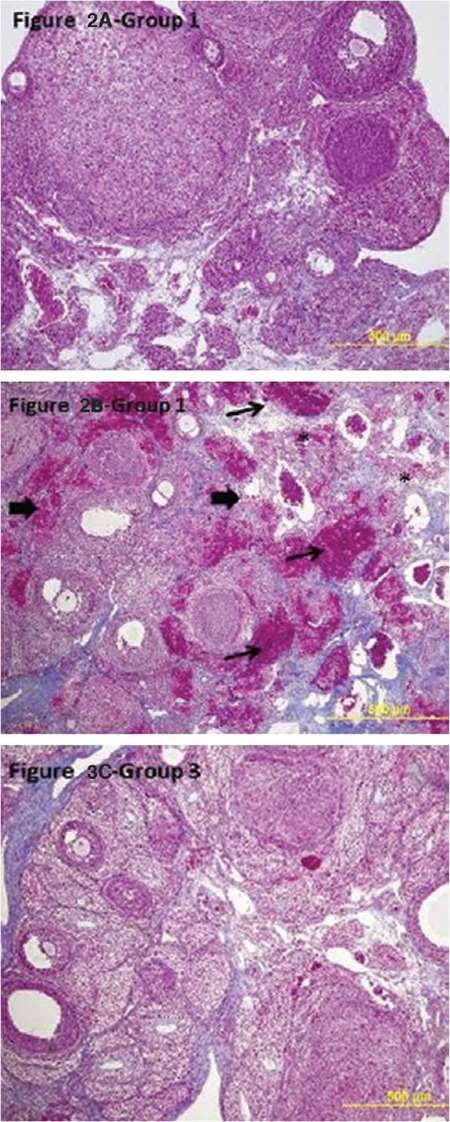
Light microscopic findings of the groups (100x, Original magnification, Masson trichrome). Ovarian sections in group 2 (I/R) showed severe damage, infiltration of polymorphonuclear leukocytes, vascular congestion, interstitial edema*, hemorrhage → → *I/R: Ischemia/reperfusion*

In the ovarian tissues of rats with I/R, normal histologic structure was deteriorated and there were pathologic findings such as interstitial edema and polymorphonuclear leukocyte infiltration. Congestion and diffuse hemorrhagic findings were also detected. The histologic structure was preserved in ovarian samples of rats in the I/R+PG group compared with those in the I/R group. In this group, less injury was noted in the follicles and the interstitial area. Edema, vascular congestion, and polymorphonuclear leukocyte infiltration were found to be decreased in the interstitial area compared with the I/R group (Figure 2).

## Discussion

Based on this experimental study, progesterone appears to play a protective role against I/R injury in ovarian torsion in rats. The mechanisms by which progesterone shows this effect are the suppression of apoptosis and decreased oxidative stress in both tissue and serum levels.

In this study, we demonstrated that progesterone shows a protective effect against ovarian I/R injury by reducing the number of apoptotic cells and this shows the anti-apoptotic effect of progesterone. Experimental studies have shown that oxidative damage mechanisms are suppressed with use of progesterone in cardiac tissue, brain tissue, and mitochondrial membrane (14). For instance, the protective effect of progesterone on neural tissue was demonstrated when progesterone was administered either 6, 24, and 48 hours after the onset of ischemia in rats with transient focal ischemia in the middle cerebral artery of the brain. The neuroprotective effect of progesterone was also investigated in rats with traumatic brain injury, and progesterone reduced edema even when it was administered 24 hours after trauma (3). Allen et al. (4) revealed the protective activity of progesterone on retinal ischemia and cerebral ischemia. Morrissy et al. (5) showed that progesterone provides a cardioprotective effect by increasing expression of the anti-apoptotic gene Bcl-xl. Sandhi et al. (6) indicated that progesterone treatment introduced renoprotective effects by reducing lipid peroxidation through increased GSH and catalase effect in rats.

We investigated the level of MDA, which is an important marker that has been efficiently used to assess ovarian reperfusion injury in previously published studies. Arikan et al. (15) showed that MDA levels significantly increased in the I/R group but then significantly decreased after tadalafil administration. Sayar et al. (16) found that MDA level increased in their I/R group but decreased in the I/R+ozone, I/R+ellegalic acid, and I/R+ozone+ellegalic acid groups. In another study, the MDA level was detected to increase in the I/R group compared with the I/R+ethyl pyruvate group (17). Taskin et al. (18) found no significant difference between tissue and serum MDA levels in sham, I/R, and I/R+2-amino ethoxy diphenyl borate (2-ABP) groups in their study with 2-APB. However, tissue MDA levels were lower in the group treated with 2-APB, although it was not statistically significant. This discrepancy might be attributed to several factors such as inadequate duration of I/R, variations in MDA measurement techniques or use of an inadequate progesterone dose. 

In this study, using progesterone in I/R injury decreased serum and tissue TOS and OSI levels but increased tissue TAS level. The results can be attributed to the anti-oxidant effects of progesterone. It has been indicated in some studies that tissue produces antioxidants excessively in response to oxidative stress in order to control unwanted ROS. For this reason, we also included TAS, TOS, and OSI parameters besides MDA to detect I/R injury. The values obtained in the present study was similar to those in the literature. In an ovarian I/R injury model, curcumin treatment caused no change in TAS levels but caused a decrease in TOS level (19). Taskin et al. (18) showed that tissue and serum TOS and OSI levels increased in the I/R group but decreased after treatment with 2-APB. 

The expression of NGAL after renal ischemia was shown to increase by Zhao et al. (20). Gong et al. (21) revealed that NGAL used the BCL2\BAX pathway in renal tubular epithelial cell apoptosis. Zang et al. (22) demonstrated that NGAL reduced apoptotic tubular cells and showed this renoprotective effect inhibiting caspase-3 activation. In the current study, the mean serum and tissue NGAL levels were statistically similar between the groups. However, tissue and serum NGAL levels increased in the I/R group compared with the sham group and decreased in the I/R+PG group, although they were not statistically significant. This finding could be due to an inadequate duration of I/R, variations in NGAL measurement techniques or the relatively small study cohort.

It was observed in a study using osajin in ovarian I/R injury that each dose of osajin reduced polymorphonuclear leukocyte infiltration at different levels (23). Borekçi et al. (24) found mononuclear cell infiltration, vascular dilatation, perivascular edema, and necrotic changes histopathologically in the I/R group in a study conducted using dehydroepiandrosterone. Haftacı et al. (25) evaluated edema, congestion, hemorrhage, and cohesion loss in their study conducted using vitamin C and selenium, and observed a reduction of cellular damage in the group administered vitamin C and selenium compared with the I/R group. Gungor et al. (26) showed in a study using omegaven that congestion, hemorrhage, and edema parameters were similar between the groups given high-dose omegaven and the sham group. In this study, we found a deterioration of normal histologic structure and pathologic findings such as interstitial edema and polymorphonuclear leukocyte infiltration in ovarian tissues of rats with I/R. Congestion and diffuse hemorrhagic findings were also found. The histologic structure was preserved in ovarian samples of rats with I/R+PG compared with the I/R group. In this group, less injury was noted in the follicles and the interstitial area. Histologic scores were found to be consistent with these findings.

In our study, the number of apoptotic cells observed as TUNEL-positive in the ovary was statistically higher in the I/R group compared with the sham group. On the other hand, the number of apoptotic cells decreased in the group given progesterone because it reflects the anti-apoptotic effects of progesterone. In an ovarian torsion  experimental study using a TUNEL assay, Taskin et al. (18) showed that apoptosis in ovarian tissue was reduced by 2-APB treatment. Sahin Ersoy et al. (27) evaluated the effects of N-acetylcysteine and enoxaparin on ovarian I/R injury. Using a TUNEL assay, the authors demonstrated that both molecules reduced the number of apoptotic cells, and found that N-acetylcysteine provided greater reduction. Gencer et al. (11) observed in a study using quercetin that TUNEL-positive cell numbers and caspase-3 values decreased in a group treated with quercetin. Progesterone has been shown to inhibit apoptosis in many studies in the literature. Espinosa-García et al. (28) demonstrated with the TUNEL method that there was a decrease in DNA fragmentation and caspase-3 level, an indirect indicator of apoptosis, in hippocampal cerebral ischemia treated with progesterone. Ishrat et al. (29) showed that progesterone used in ischemic brain injury treatment reduced apoptosis in cerebral ischemia using phosphoinositol kinase/kinase B pathway.

There are some limitations to this study. For example, the duration of the ischemia is controversial. We used a similar duration to that used in experimental models in the literature. Another limitation is that we did not use different doses for progesterone. Also, the number of animals in each group was limited. There is another controversial point, not every experimental model identically reflects human physiology. Nevertheless, there are some strengths to this study. We evaluated both serum and tissue oxidation parameters and used the TUNEL method to determine the apoptotic cell number. The advantage of progesterone is that it is not toxic for human physiology and is generally used for gynecologic and obstetric conditions.

In our experiments, we found that progesterone inhibited the apoptosis process and decreased the oxidative burden in torsioned rat ovaries. The effect of progesterone needs to be tested in preclinical studies to better understand its role in protecting the ovary from ischemia and reperfusion injury in the management of ovarian torsion.

## Figures and Tables

**Table 1:**
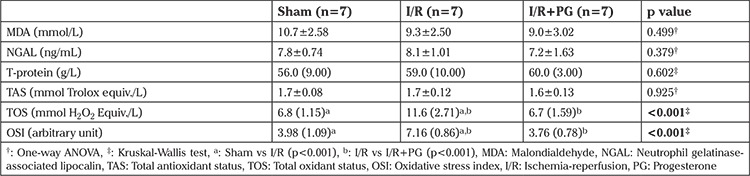
Biochemical (serum) measurements according to groups

**Table 2:**
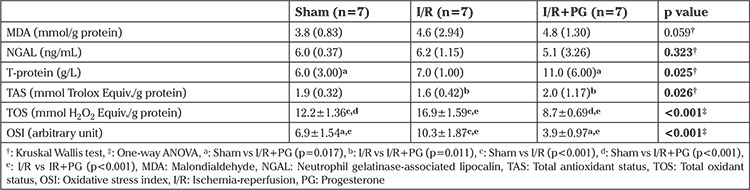
Biochemical (tissue) measurements according to groups

**Graphic 1:**
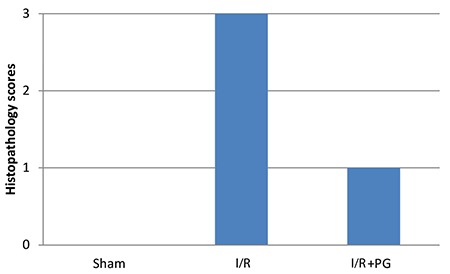
Histopathologic score in groups *I/R: Ischemia/reperfusion, PG: Progesterone*
